# Development and validation of web-based nomograms for predicting survival status in patients with intrahepatic cholangiocarcinoma depending on the surgical status: a SEER database analysis

**DOI:** 10.1038/s41598-024-52025-3

**Published:** 2024-01-18

**Authors:** Yao-Ge Liu, Shi-Tao Jiang, Jun-Wei Zhang, Lei Zhang, Hai-Tao Zhao, Xin-Ting Sang, Xin Lu, Yi-Yao Xu

**Affiliations:** Department of Liver Surgery, Peking Union Medical College Hospital, Chinese Academy of Medical Sciences and Peking Union Medical College (CAMS and PUMC), Beijing, China

**Keywords:** Oncology, Risk factors

## Abstract

This study aimed to develop and validate prognostic nomograms that can estimate the probability of 1-, 3- and 5-year overall survival (OS) as well as cancer-specific survival (CSS) for Intrahepatic cholangiocarcinoma (ICCA) patients. Clinical data of 1446 patients diagnosed with ICCA between 2010 and 2017 from the Surveillance, Epidemiology, and End Results (SEER) database were analyzed. In both the OS and the CSS group, the training cohort and validation cohort were divided into a 7:3 ratio. Age, sex, AJCC T stage, AJCC N stage, AJCC M stage, surgical status, and tumor grade were selected as independent prognostic risk factors to build the nomograms. To compare the efficacy of predicting 1-, 3-, and 5-year OS and CSS rates of the nomogram with the 8th edition of the American Joint Committee on Cancer (AJCC) staging system, we evaluated the Harrell’s index of concordance (C-index), area under the receiver operating characteristic curve (AUC) and decision curve analysis (DCA) in both cohorts. The results showed the nomogram for 1-, 3-, and 5-year OS and CSS prediction performed better than the AJCC staging system. In the subgroup analysis for patients could not receive surgery as the primary treatment. We developed two nomograms for predicting the 1-, and 2-year OS and CSS rates following the same analysis procedure. Results indicate that the performance of both nomograms, which contained sex, AJCC T stage, AJCC M stage, chemotherapy, and tumor grade and prognostic factors, was also superior to the AJCC staging system. Meanwhile, four dynamic network-based nomograms were published. The survival analysis showed the survival rate of patients classified as high-risk based on the nomogram score was significantly lower compared to those categorized as low-risk (P < 0.0001). Finally, accurate and convenient nomograms were established to assist clinicians in making more personalized prognosis predictions for ICCA patients.

## Introduction

Intrahepatic cholangiocarcinoma (ICCA) is a highly lethal tumor with a 5‐year overall survival (OS) near 9%^1^. It remains the second most prevalent primary liver cancer, which is derived from epithelial cells of the second‐order bile ducts and represents approximately 20% of all hepatic malignancies^2^. In the past 4 decades, the prevalence of ICCA in the U.S. has been increasing rapidly from 0.44 to 1.18 cases per million^3^. However, compared with perihilar cholangiocarcinoma (pCC) or distal cholangiocarcinoma (dCC), which were both anatomic subtypes of cholangiocarcinoma, the early stage of ICCA was difficult to diagnose due to the asymptomatic clinical characters^4^. Most patients diagnosed with ICCA were at the advanced stage and only 20–30% of them were able to undergo complete surgical resection which remained the only potential therapy^5^. For patients in the advanced stage of ICCA, the chemotherapy regimen of gemcitabine plus cisplatin has been recommended as the first‐line therapy^6^. In addition, evidence regarding the survival benefits of radiation therapy, whether used alone or in combination, in the treatment of advanced-stage ICCA patients, is continuously accumulating^7^.

At present, the most commonly accepted survival prediction method for ICCA patients is the 8th edition American Joint Committee on Cancer (AJCC) staging system. However, it only analyzed factors including the local extension of the primary tumor, lymph node, and distant metastasis which neglected other factors such as age, sex, histological grade, and treatment status. Hence, it is necessary to incorporate various prognostic factors into one predictive system which would enable researchers to predict the prognosis of patients with greater accuracy.

Nomogram is a widely used tool for clinical decision-making, which could calculate the probability of clinical events by considering the prognostic weight of each factor and presenting the results visually^8^. Recent research endeavors have focused on developing nomograms as predictive tools for assessing the prognosis of patients with ICCA from different aspects. Some studies focused on predicting the overall survival (OS) of ICCA patients^9–11^, while some tried to predict the cancer-specific survival (CSS) of ICCA patients after surgery^12,13^. Liu et al. tried to construct a nomogram for predicting CSS in ICCA patients but failed due to the relatively small sample size (n = 189)^14^. However, insufficient research has been conducted on a comprehensive cohort of ICCA patients, including those who were ineligible for surgical treatment, resulting in a dearth of predictive models for estimating CSS of ICCA patients of all stages. Based on the current research status, we aimed to conduct a more comprehensive analysis of survival status prediction for patients with ICCA, including OS and CSS analyses. Furthermore, we conducted a subgroup analysis and discussion based on the overall analysis results.

In this study, we extracted independent prognostic factors obtained from the Surveillance, Epidemiology, and End Results (SEER) database to develop and validate two nomograms that can accurately predict OS and CSS in patients diagnosed with ICCA of different stages. According to the nomogram analysis results for OS and CSS, we performed a subgroup analysis according to the surgical status. Additionally, two separate nomograms were developed for patients who could not undergo surgery for primary treatment. In addition, by comparing the nomogram with the 8th AJCC staging system, we expect to demonstrate the model we established with greater performance.

## Materials and methods

### Study population and data extraction

We selected patients diagnosed with ICCA from the Incidence—SEER Research Plus Data, 18 Registries, Nov 2020 Sub (2000–2018) database. The data were acquired from the SEER*Stat software version 8.4.1. The inclusion criteria were as follows: (1) the International Classification of Diseases for Oncology, Third Edition (ICD-O-3) code was 8160/3: cholangiocarcinoma; (2) Primary Site-Labeled: C22.1-Intrahepatic bile duct; (3) Year of Diagnosis: “2010”, “2011”, “2012”, “2013”, “2014”, “2015”, “2016”, “2017”. Through preliminary screening, we obtained clinical data from 7401 patients. The exclusion criteria were as follows: (1) age of diagnosis below 20; (2) missing data of race, tumor (T) staging, lymph node (N) staging, and metastasis (M) staging of the AJCC staging system, surgical status, tumor grade, and tumor size; (3) ICCA not as the only or first primary tumor; (4) CSS less than 1 month. A total of 1446 patients meeting the criteria were included in the study.

**Figure 1:**
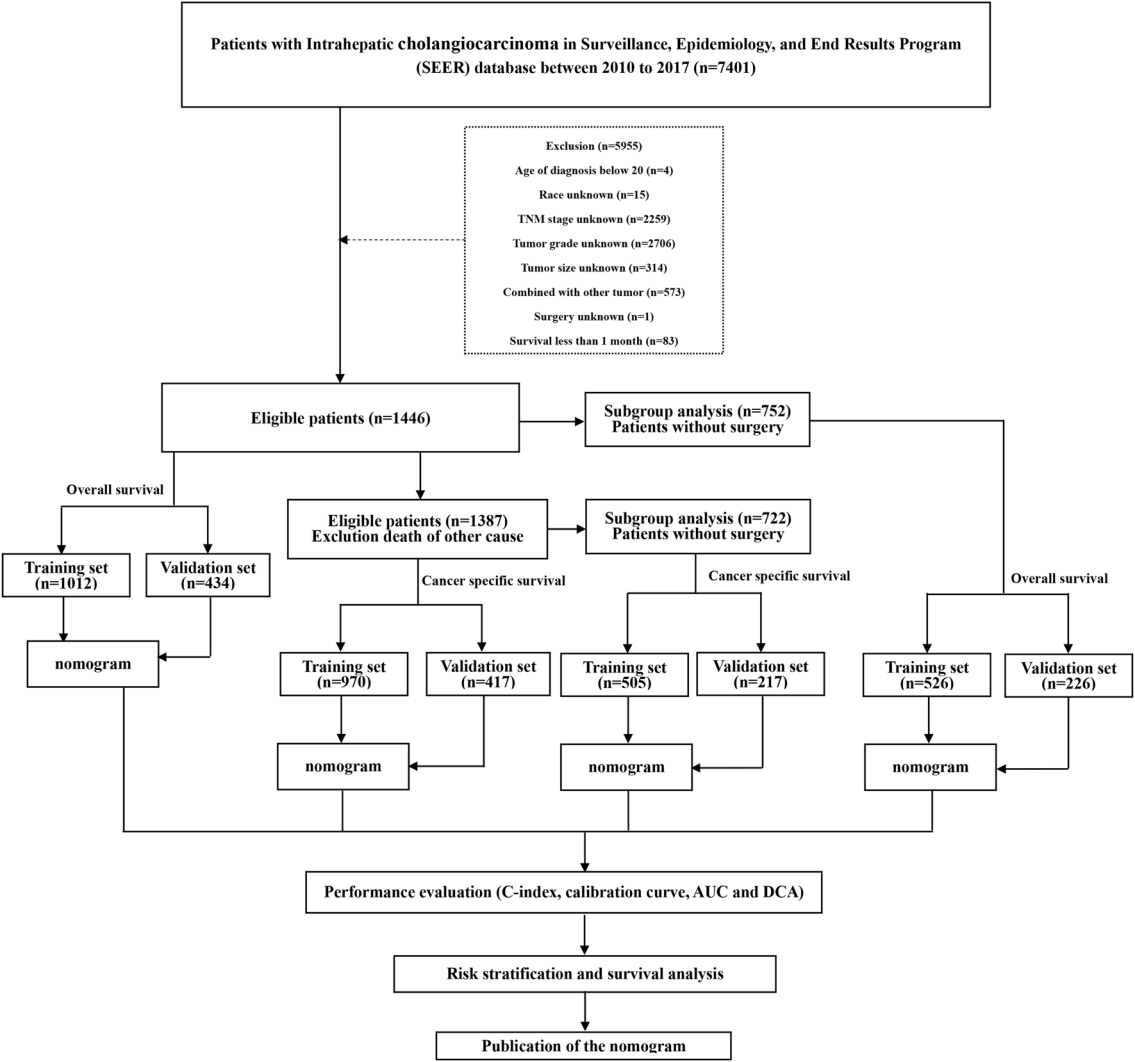
Flowchart of screening criteria and key steps in the study.

Based on the 1446 eligible patients, we further conducted a subgroup survival analysis based on the patient's cause of death, specifically whether it was due to the cancer, and whether the patient underwent surgical treatment. A total of four nomograms were established and validated to predict the survival status of ICCA patients. The overview of the research process is shown in Fig. 1.

### Study variables

Clinical variables of each patient were obtained including year of diagnosis, age at diagnosis, sex, race, tumor size, tumor grade, surgical status, AJCC 7th edition of TNM stage, survival months, cause of death, chemotherapy, and radiation status. The tumor staging system was translated from the 7th edition AJCC system which was available for patients from 2010 to 2017 to the 8th edition based on tumor characteristics^15^. The age variable was divided into four groups, namely (1) below 50 years old; (2) 50–64 years old; (3) 65–79 years old; (4) no less than 80 years old. Tumor size was divided into two groups: diameter no more than 5 cm and over 5 cm. OS was defined as the time from diagnosis to death or the last follow-up, and CSS excluded the death of other causes.

### Statistical analysis

R (version 4.2.1) was the primary analytical software utilized in this study. Two-tailed P values < 0.05 were considered statistically significant. Each analysis group was divided into the training cohortand validation cohortwith a proportion of 7:3 randomly using the “Random Samples and Permutations” function in R^14,16^. The training cohort was utilized for model construction, while the validation cohort was employed for model validation. Continuous data were reported as the median with interquartile range (IQR), while categorical data were presented as frequencies (proportions) and compared using the Chi-square test. In the univariable Cox proportional hazards regression analyses, the Wald test was used and significance thresholds below 0.05 was considered to be statistically significant. Univariable Cox proportional hazards regression analyses were performed to screen statistically significant variables for further multivariate Cox proportional hazards regression analyses. Seven independent prognostic factors were selected for nomogram construction for predicting 1-, 3- and 5-year OS and CSS rates in ICCA patients based on Kaplan–Meier analyses using the *survival* package of R. Based on the same analytical method, five independent prognostic factors were screened out for nomogram construction in the subgroup analysis for predicting 1-, and 2-year OS and CSS rates in ICCA patients.

To evaluate the performance of the nomogram, the discriminative power was assessed using the Harrell concordance index (C-index) through internal validation and external validation using the validation cohort. The R packages *Hmisc* and *rms* were employed, and bootstrap resampling with 1000 resamples was performed^9^. The agreement between predictions and observations was evaluated by the calibration curve using R package *rms*^17^. Compared to the AJCC 8th TNM staging system as a reference, the time-dependent area under the ROC curves (AUC) using R package *survivalROC*^18^ and decision curve analysis (DCA) using R package *rmda*^19^ were employed to evaluate the clinical utility and net benefit of the nomogram. In addition, based on the scores derived from the nomogram constructed using the training cohort, patients from two cohorts were divided into high-risk and low-risk groups in a 1:1 ratio accordingly. To assess the risk stratification effect of the nomogram, survival analysis was conducted utilizing Kaplan–Meier (KM) curves and Cox proportional hazard models. Finally, the R package *DynNom* was utilized to build a web-based nomogram on the Shiny application platform^20^.

### Ethical approval

The author Y-GL has gotten access to the SEER database and signed the “Surveillance, Epidemiology, and End Results Program Data Use Agreement”. This study obtained clinical data from the SEER database, where informed consent from patients was not required.

## Results

### Demographic and clinical characteristics

A total of 1446 patients diagnosed with ICCA were included from 2010 to 2017 to ensure a follow-up time of no less than 5 years. In the OS analysis group, there were 1446 eligible patients. The training (n = 1012) and validation (n = 434) cohort was randomly generated from the whole population with a proportion of 7:3. In the total cohort of ICCA patients, the majority of patients were between 50 to 80 years old (80.7%) and white (78.1%). The majority of patients were diagnosed with T1 and T2 stages (77.0%), with no lymph nodes (67.0%) or distant metastasis (75.9%). Out of the total cohort, only 13 patients had pathological grade IV, making up a mere 0.9% of the proportion. Accordingly, the groups with pathological grades IV and III were consolidated into a single category. Regarding treatment, surgical intervention was performed in 48.0% of the patients, while the majority (61.5%) underwent chemotherapy. Radiotherapy, on the other hand, was administered to only 16.0% of the patients. The training set and validation set exhibit statistically significant differences in Grade distribution (P = 0.013). Apart from this, the other baseline characteristics between the two cohorts were balanced (Table 1).

**Table 1:** Demographic and clinical characteristics of patients with Intrahepatic cholangiocarcinoma in the OS group. *IQR* interquartile range.

Characteristic	Overall	Training cohort	Validation cohort	P value
Total patients, n	1446	1012	434	
Age, n (%)				0.620
< 50	170 (11.8%)	123 (12.2%)	47 (10.8%)	
50–64	541 (37.4%)	384 (37.9%)	157 (36.2%)	
65–79	626 (43.3%)	427 (42.2%)	199 (45.9%)	
≥ 80	109 (7.5%)	78 (7.7%)	31 (7.1%)	
Sex, n (%)				0.837
Male	747 (51.7%)	521 (51.5%)	226 (52.1%)	
Female	699 (48.3%)	491 (48.5%)	208 (47.9%)	
Race, n (%)				0.361
White	1129 (78.1%)	786 (77.7%)	343 (79.0%)	
Black	119 (8.2%)	90 (8.9%)	29 (6.7%)	
Other	198 (13.7%)	136 (13.4%)	62 (14.3%)	
AJCC T stage, n (%)				0.930
T1	452 (31.3%)	316 (31.2%)	136 (31.3%)	
T2	661 (45.7%)	463 (45.8%)	198 (45.6%)	
T3	221 (15.3%)	152 (15.0%)	69 (15.9%)	
T4	112 (7.7%)	81 (8.0%)	31 (7.1%)	
AJCC N stage, n (%)				0.528
N0	969 (67.0%)	673 (66.5%)	296 (68.2%)	
N1	477 (33.0%)	339 (33.5%)	138 (31.8%)	
AJCC M stage, n (%)				0.481
M0	1097 (75.9%)	773 (76.4%)	324 (74.7%)	
M1	349 (24.1%)	239 (23.6%)	110 (25.3%)	
Grade, n (%)				0.013
I	145 (10.0%)	111 (11.0%)	34 (7.8%)	
II	686 (47.4%)	456 (45.1%)	230 (53.0%)	
III + IV	615 (42.5%)	445 (44.0%)	170 (39.2%)	
Tumor size, n (%)				0.730
≤ 5 cm	506 (35.0%)	357 (35.3%)	149 (34.3%)	
> 5 cm	940 (65.0%)	655 (64.7%)	285 (65.7%)	
Surgery, n (%)				0.179
No	752 (52.0%)	538 (53.2%)	214 (49.3%)	
Yes	694 (48.0%)	474 (46.8%)	220 (50.7%)	
Chemotherapy, n (%)				0.247
No	557 (38.5%)	380 (37.5%)	177 (40.8%)	
Yes	889 (61.5%)	632 (62.5%)	257 (59.2%)	
Radiotherapy, n (%)				0.954
No	1214 (84.0%)	850 (84.0%)	364 (83.9%)	
Yes	232 (16.0%)	162 (16.0%)	70 (16.1%)	
Survival, median (IQR)	15 (6.0, 28.00)	15 (6.0, 28.00)	16 (7.0, 28.50)	0.505

Excluding 59 patients who died due to other causes of death, a total of 1387 patients were included in the CSS analysis. The training (n = 970) and validation (n = 417) cohort was also distributed in a 7:3 ratio of populations. In terms of demographic characteristics, the CSS group and OS group exhibited similar compositions. The majority of the patients were white (77.9%) and between 50 to 80 years old (80.8%). 1065 patients (76.8%) were diagnosed with T1 and T2 stages. The majority (52.1%) of patients have not undergone surgery and only 16.1% of patients underwent radiotherapy. The proportion of patients receiving chemotherapy was higher in the training set with statistical significance (P = 0.014). The rest baseline characteristics between the two cohorts were balanced (Table 2).

**Table 2:** Demographic and clinical characteristics of patients with Intrahepatic cholangiocarcinoma in the CSS group. *IQR* interquartile range.

Characteristic	Overall	Training cohort	Validation cohort	P value
Total patients, n	1387	970	417	
Age, n (%)				0.816
< 50	170 (12.3%)	122 (12.6%)	48 (11.5%)	
50–64	527 (38.0%)	361 (37.2%)	166 (39.8%)	
65–79	593 (42.8%)	418 (43.1%)	175 (42.0%)	
≥ 80	97 (7.0%)	69 (7.1%)	28 (6.7%)	
Sex, n (%)				0.876
Male	714 (51.5%)	498 (51.3%)	216 (51.8%)	
Female	673 (48.5%)	472 (48.7%)	201 (48.2%)	
Race, n (%)				0.383
White	1081 (77.9%)	749 (77.2%)	332 (79.6%)	
Black	116 (8.4%)	80 (8.2%)	36 (8.6%)	
Other	190 (13.7%)	141 (14.5%)	49 (11.8%)	
AJCC T stage, n (%)				0.227
T1	422 (30.4%)	304 (31.3%)	118 (28.3%)	
T2	643 (46.4%)	433 (44.6%)	210 (50.4%)	
T3	216 (15.6%)	159 (16.4%)	57 (13.7%)	
T4	106 (7.6%)	74 (7.6%)	32 (7.7%)	
AJCC N stage, n (%)				0.315
N0	921 (66.4%)	636 (65.6%)	285 (68.3%)	
N1	466 (33.6%)	334 (34.4%)	132 (31.7%)	
AJCC M stage, n (%)				0.777
M0	1048 (75.6%)	735 (75.8%)	313 (75.1%)	
M1	339 (24.4%)	235 (24.2%)	104 (24.9%)	
Grade, n (%)				0.350
I	139 (10.0%)	98 (10.1%)	41 (9.8%)	
II	655 (47.2%)	446 (46.0%)	209 (50.1%)	
III + IV	593 (42.8%)	426 (43.9%)	167 (40.0%)	
Tumor size, n (%)				0.261
≤ 5 cm	476 (34.3%)	342 (35.3%)	134 (32.1%)	
> 5 cm	911 (65.7%)	628 (64.7%)	283 (67.9%)	
Surgery, n (%)				0.200
No	722 (52.1%)	494 (50.9%)	228 (54.7%)	
Yes	665 (47.9%)	476 (49.1%)	189 (45.3%)	
Chemotherapy, n (%)				0.014
No	520 (37.5%)	384 (39.6%)	136 (32.6%)	
Yes	867 (62.5%)	586 (60.4%)	281 (67.4%)	
Radiotherapy, n (%)				0.242
No	1163 (83.9%)	806 (83.1%)	357 (85.6%)	
Yes	224 (16.1%)	164 (16.9%)	60 (14.4%)	
Survival, median (IQR)	15 (7.0, 28.00)	15 (6.0, 29.00)	16 (7.0, 27.00)	0.579

### Variables screening and nomogram construction

Prior to conducting the univariable Cox regression analysis for each group, we employed Spearman's correlation to verify the absence of collinearity among the screened variables (Supplementary Fig. 1).

In the OS group, we selected eight independent prognostic factors based on the univariable Cox proportional hazards regression analyses in the training cohort: age no less than 65 years old (hazard ratio [HR] = 1.416/2.073, P < 0.01), male (HR = 1.207, P = 0.011), AJCC T2/T3/T4 (HR = 1.651/1.923/1.807, P < 0.001), AJCC N1 (HR = 1.746, P < 0.001), AJCC M1 (HR = 2.407, P < 0.001), tumor grade III + IV (HR = 1.746, P < 0.001), tumor size over 5 cm (HR = 1.458, P < 0.001) and receiving surgery (HR = 0.311, P < 0.001). In the next step, multivariable Cox proportional hazards regression analyses were conducted based on these eight prognostic factors. As a result, seven independent prognostic factors were identified, which showed a significant association with OS in patients with ICCA (Table 3). No significant association between tumor size and OS was revealed in the multivariable Cox regression analysis (HR = 1.048, P = 0.575). In addition, chemotherapy (HR = 0.935, P = 0.380) and radiotherapy (HR = 0.911, P = 0.353) were not prognostic factors for OS in ICCA patients.

**Table 3:** Univariable and multivariable Cox analyses on variables for the prediction of overall survival of intrahepatic cholangiocarcinoma patients. *HR* hazard ratio,* CI* confidence interval, *P < 0.05, **P < 0.01, ***P < 0.001.

Variables	Univariable Cox analysis		Multivariable Cox analysis	
	HR (95% CI)	P value	HR (95% CI)	P value
Age				
< 50	Reference		Reference	
50–64	1.251 (0.980–1.595)	0.072	1.257 (0.983–1.607)	0.068
65–79	1.416 (1.113–1.800)	0.005**	1.557 (1.220–1.986)	< 0.001***
≥ 80	2.073 (1.502–2.862)	< 0.001***	2.421 (1.724–3.399)	< 0.001***
Sex				
Female	Reference		Reference	
Male	1.207 (1.044–1.394)	0.011*	1.228 (1.062–1.421)	0.006**
Race				
White	Reference			
Black	1.094 (0.849–1.409)	0.488		
Other	1.020 (0.825–1.261)	0.845		
AJCC T stage				
T1	Reference		Reference	
T2	1.651 (1.383–1.971)	< 0.001***	1.388 (1.153–1.670)	< 0.001***
T3	1.923 (1.534–2.410)	< 0.001***	1.747 (1.384–2.206)	< 0.001***
T4	1.807 (1.367–2.389)	< 0.001***	1.923 (1.448–2.553)	< 0.001***
AJCC N stage				
N0	Reference		Reference	
N1	1.746 (1.502–2.030)	< 0.001***	1.353 (1.154–1.588)	< 0.001***
AJCC M stage				
M0	Reference		Reference	
M1	2.407 (2.049–2.827)	< 0.001***	1.461 (1.219–1.751)	< 0.001***
Grade				
I	Reference		Reference	
II	1.134 (0.877–1.466)	0.338	1.153 (0.889–1.495)	0.285
III + IV	1.746 (1.354–2.251)	< 0.001***	1.623 (1.255–2.099)	< 0.001***
Tumor size				
≤ 5 cm	Reference		Reference	
> 5 cm	1.458 (1.248–1.705)	< 0.001***	1.048 (0.890–1.235)	0.575
Surgery				
No	Reference		Reference	
Yes	0.311 (0.266–0.364)	< 0.001***	0.409 (0.343–0.488)	< 0.001***
Chemotherapy				
No	Reference			
Yes	0.935 (0.804–1.087)	0.380		
Radiotherapy				
No	Reference			
Yes	0.911 (0.747–1.110)	0.353		

In the CSS group, by employing univariable and multivariable Cox regression analyses, the same seven prognostic factors were identified: age, sex, AJCC T stage, AJCC N stage, AJCC M stage, surgery status, and tumor grade. The detailed information on univariable and multivariable Cox regression analyses in the CSS group is shown in Table 4.

**Table 4:** Univariable and multivariable Cox analyses on variables for the prediction of cancer-specific survival of intrahepatic cholangiocarcinoma patients. *HR* hazard ratio, *CI* confidence interval, *P < 0.05, **P < 0.01, ***P < 0.001.

Variables	Univariable Cox analysis		Multivariable Cox analysis	
	HR (95% CI)	P value	HR (95% CI)	P value
Age				
< 50	Reference		Reference	
50–64	1.212 (0.945–1.533)	0.129	1.210 (0.943–1.553)	0.135
65–79	1.255 (0.983–1.602)	0.068	1.335 (1.044–1.708)	0.022*
≥ 80	1.836 (1.307–2.578)	< 0.001***	2.159 (1.527–3.052)	< 0.001***
Sex				
Female	Reference		Reference	
Male	1.190 (1.024–1.383)	0.024*	1.179 (1.011–1.374)	0.035*
Race				
White	Reference			
Black	1.058 (0.805–1.391)	0.685		
Other	0.930 (0.750–1.155)	0.512		
AJCC T stage				
T1	Reference		Reference	
T2	1.717 (1.427–2.064)	< 0.001***	1.400 (1.155–1.696)	< 0.001***
T3	2.052 (1.631–2.582)	< 0.001***	1.837 (1.450–2.327)	< 0.001***
T4	1.822 (1.350–2.459)	< 0.001***	1.917 (1.417–2.595)	< 0.001***
AJCC N stage				
N0	Reference		Reference	
N1	1.940 (1.659–2.268)	< 0.001***	1.324 (1.122–1.563)	< 0.001***
AJCC M stage				
M0	Reference		Reference	
M1	2.840 (2.406–3.353)	< 0.001***	1.520 (1.259–1.833)	< 0.001***
Grade				
I	Reference		Reference	
II	1.191 (0.900–1.576)	0.222	1.152 (0.868–1.527)	0.328
III + IV	1.834 (1.389–2.421)	< 0.001***	1.621 (1.223–2.149)	< 0.001***
Tumor size				
≤ 5 cm	Reference		Reference	
> 5 cm	1.595 (1.354–1.880)	< 0.001***	1.094 (0.920–1.300)	0.310
Surgery				
No	Reference		Reference	
Yes	0.284 (0.242–0.334)	< 0.001***	0.373 (0.311–0.449)	< 0.001***
Chemotherapy				
No	Reference			
Yes	1.040 (0.889–1.217)	0.622		
Radiotherapy				
No	Reference			
Yes	0.904 (0.741–1.104)	0.322		

**Figure 2:**
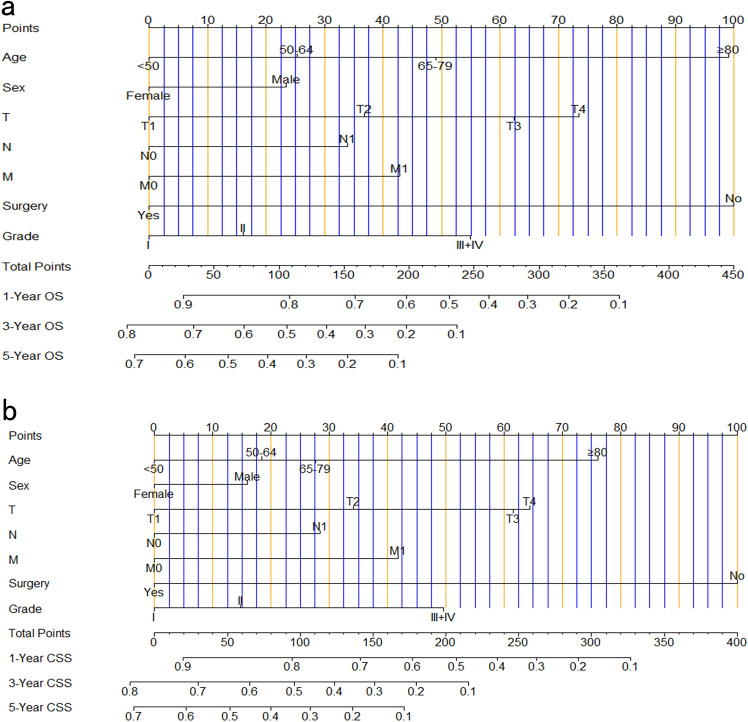
(**a**) Nomogram predicting 1-, 3- and 5-year OS of patients with ICCA. (**b**) Nomogram predicting 1-, 3- and 5-year CSS of patients with ICCA. The total points were added up from the corresponding predictors, and through the vertical correspondence relationship, we could get the predicted probability of 1-, 3- and 5-year survival.

Based on the seven prognostic factors and utilizing the training cohort as the data source, we developed two nomograms using the multivariate Cox prognostic model for ICCA patients which enabled the prediction of 1-, 3-, and 5-year OS and CSS rates (Fig. 2). For each patient, every independent predictor with different weights has its corresponding points on the first line. The total points of a patient could be acquired by adding up the points of each factor, which corresponded vertically to the 1-, 3-, and 5-year OS and CSS rates at the bottom lines.

### Nomogram validation

**Figure 3:**
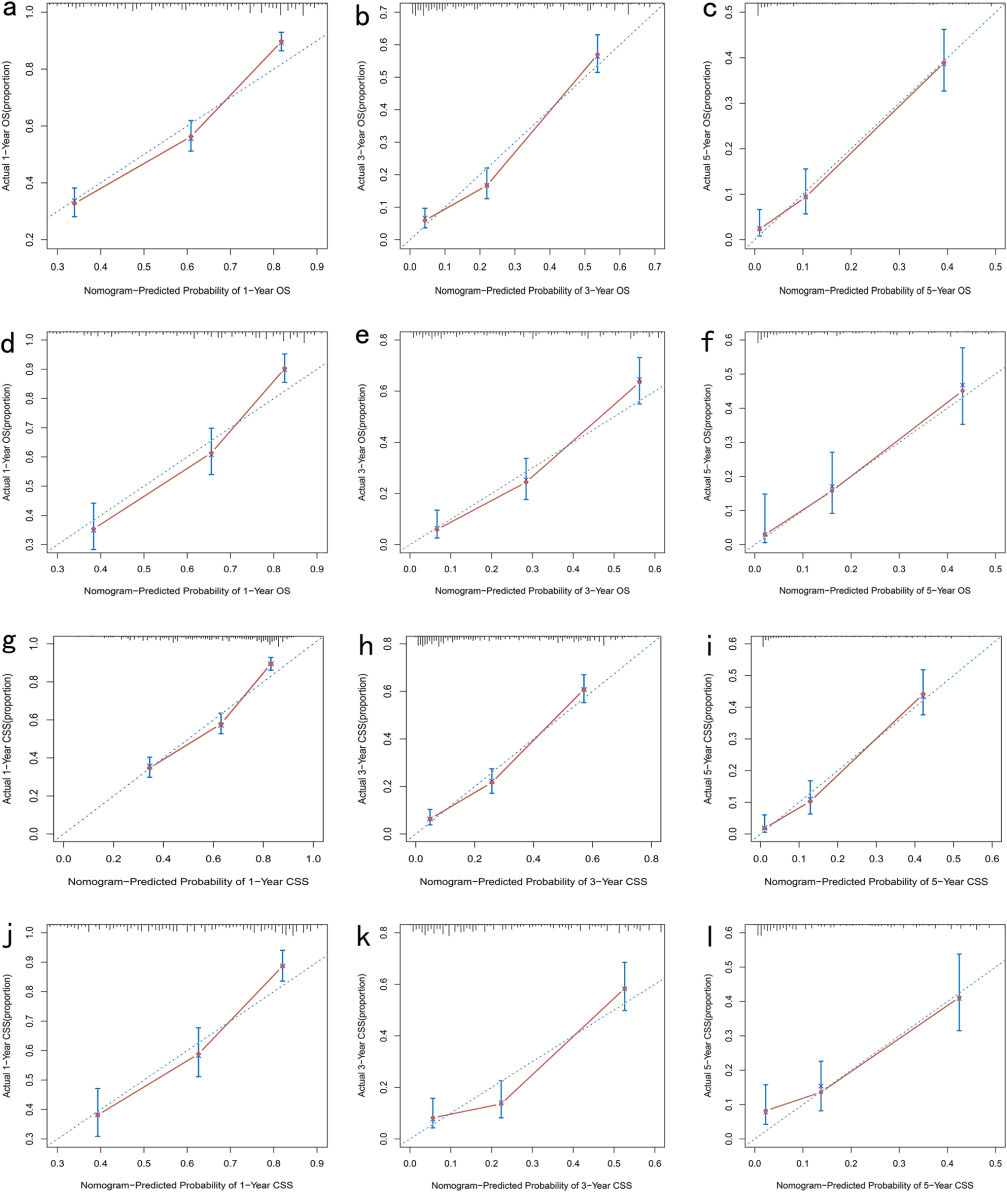
Calibration curves of the nomogram for the 1-, 3-, and 5-year OS prediction of patients with ICCA in the training cohort (**a**–**c**) and the validation cohort (**d**–**f**). Calibration curves of the nomogram for the 1-, 3-, and 5-year CSS prediction of patients with ICCA in the training cohort (**g**–**i**) and the validation cohort (**j**–**l**).

In the OS group, the C-index of the nomogram in the training and validation cohorts were 0.719 (0.695–0.743) and 0.723 (0.685–0.760) respectively. As a comparison, the C-index of the 8th AJCC staging was 0.648 (0.624–0.671) and 0.638 (0.601–0.674) in the training and validation cohorts. In the CSS group, The C-index of the nomogram in the training and validation cohorts were 0.726 (0.701–0.751) and 0.711 (0.673–0.749). In comparison, the C-index of the 8th AJCC staging was 0.663 (0.639–0.687) and 0.617 (0.580–0.654) in the training and validation cohorts. Furthermore, calibration curves of 1-, 3-, and 5-year OS and CSS were established in both cohorts (Fig. 3a–l). In the calibration plot, a model's observed and predicted probabilities depicted by a dashed line that precisely aligns with the diagonal slash on the plot would be considered highly efficient. In the training and validation cohorts, the 1-, 3-, and 5-year OS and CSS rates predicted by the nomogram were in good agreement with survival status.

**Figure 4:**
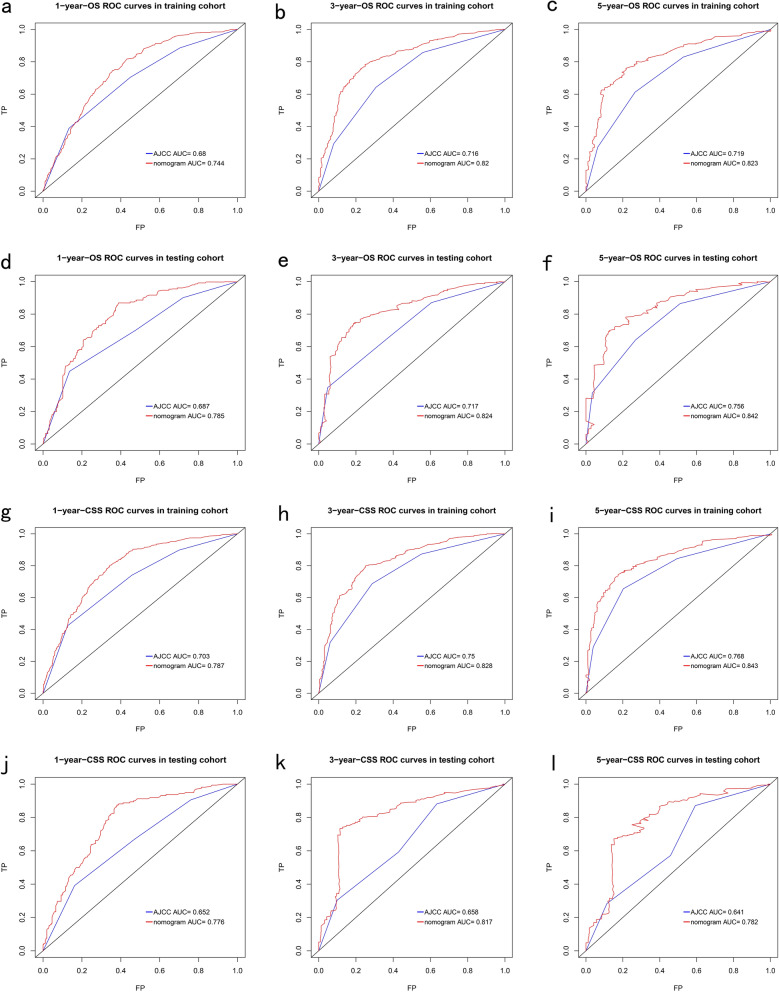
ROC curves of both the nomogram and the 8th AJCC TNM staging system in the training cohort (**a**–**c**) and the validation cohort (**d**–**f**) for 1-, 3-, and 5-year OS prediction. ROC curves of both the nomogram and the 8th AJCC TNM staging system in the training cohort (**g**–**i**) and the validation cohort (**j**–**l**) for 1-, 3-, and 5-year CSS prediction.

To compare the performance of the nomogram with the 8th AJCC staging system, the time-dependent ROC curves and DCA curves were plotted. Notably, all of these AUC values surpass the corresponding AUC values of the AJCC stage in both the OS and CSS group, the AUC of the nomogram and the AJCC stage could be observed in Fig. 4a–l. In the OS group, the nomogram consistently exhibits an AUC greater than 0.74 when predicting the 1-, 3-, and 5-year OS rates. While in the CSS group, the nomogram accordingly exhibits an AUC greater than 0.75. More parameters of the nomogram including the positive predictive value for death at 1-, 3-, and 5-year, sensitivity, and specificity at the cut-off point accordingly are listed in Table 5. The result suggested that the nomogram demonstrated favorable discrimination capabilities.

**Table 5:** Parameters of the ROC curves for the prediction of OS and CSS of intrahepatic cholangiocarcinoma patients. *AUC* areas under curve, *Se* sensitivity, *Sp* specificity, *PPV* positive predictive value.

Training cohort	AUC	Se/Sp	PPV (%)	Validation cohort	AUC	Se/Sp	PPV (%)
For 1-year OS	0.744	76.67%/63.67%	57.33	For 1-year OS	0.786	84.97%/63.26%	56.85
For 3-year OS	0.793	78.87%/68.42%	87.06	For 3-year OS	0.826	72.59%/87.50%	92.66
For 5-year OS	0.800	74.70%/72.73%	92.80	For 5-year OS	0.841	71.84%/89.66%	96.12
For 1-year CSS	0.787	82.14%/65.13%	58.72	For 1-year CSS	0.776	87.33%/60.56%	56.27
For 3-year CSS	0.828	79.97%/75.86%	88.49	For 3-year CSS	0.817	73.85%/85.25%	93.20
For 5-year CSS	0.843	74.59%/83.05%	94.79	For 5-year CSS	0.782	68.61%/95.45%	98.35

**Figure 5:**
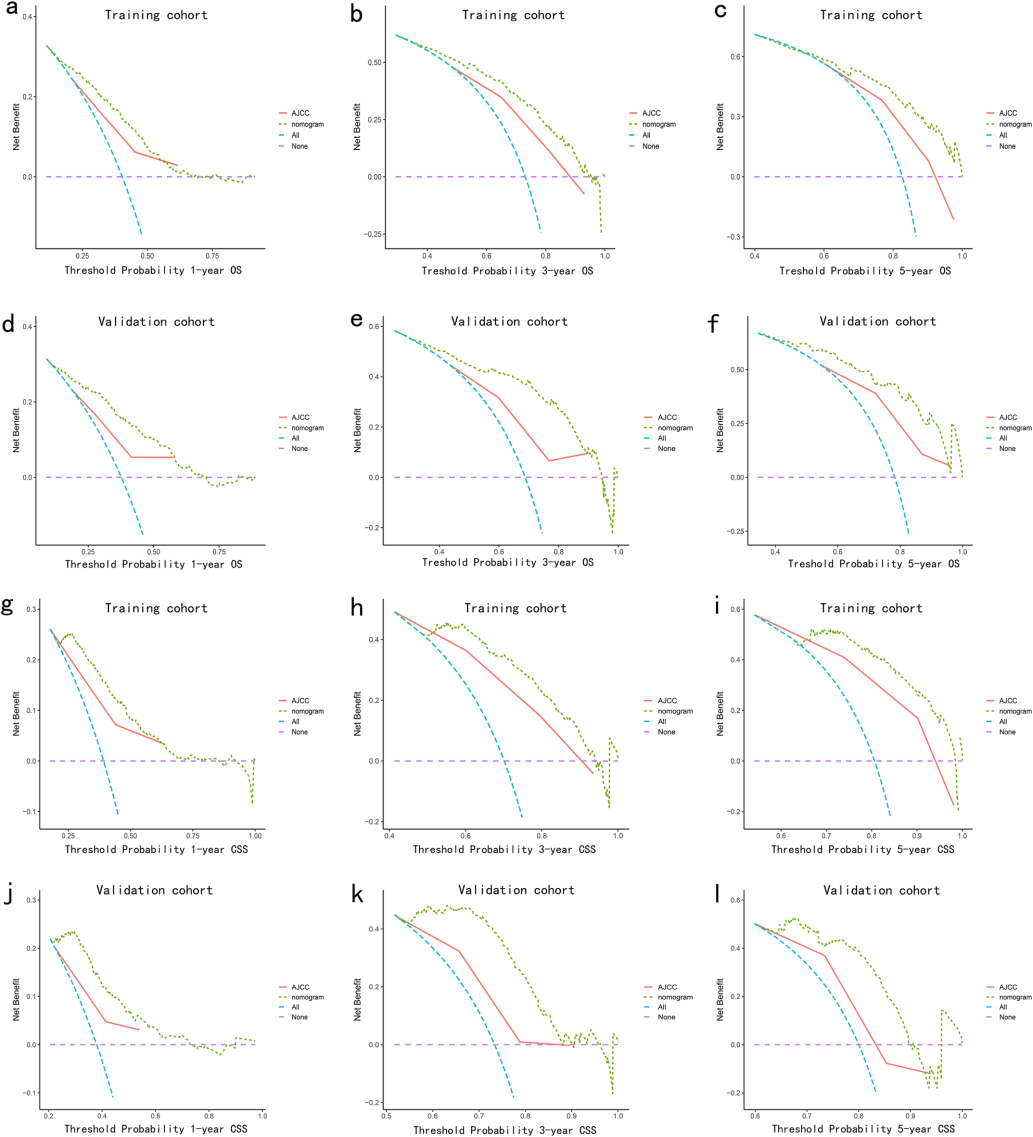
Decision curves of both the nomogram and the 8th AJCC TNM staging system in the training cohort (**a**–**c**) and the validation cohort (**d**–**f**) for 1-, 3-, and 5-year OS prediction. Decision curves of both the nomogram and the 8th AJCC TNM staging system in the training cohort (**g**–**i**) and the validation cohort (j–l) for 1-, 3-, and 5-year CSS prediction. The x-axis is the threshold probability, and the y-axis is the net benefit rate. The horizontal dashed line indicates 0 net benefits when all patients with ICCA are not treated. The smooth diagonal line indicates when all patients with ICCA are treated regardless of the predictive model.

In addition, we performed DCA analyses in both cohorts to demonstrate the net benefit of the nomogram compared to the AJCC stage (Fig. 5a–l). The DCA analysis can reveal the variation in net benefit as the threshold probability changes based on the model's predicted values^21^. For instance, in Fig. 5a, if the threshold that one patient had the possibility of 45% dying within 1 year (at the 45% risk threshold), the net benefit was higher in the nomogram model than in the AJCC staging system. In other words, the use of a nomogram for prognosis analysis and subsequent treatment decisions may yield greater net benefits compared to decisions based on the AJCC staging system from wider thresholds. The decisions involved comprehensive and proactive treatment measures for patients, including surgery, aiming to improve both OS and CSS. As a result, the developed nomogram exhibited a greater net benefit when predicting the 1-, 3-, and 5-year OS and CSS rates compared to the 8th AJCC TNM staging system.

Above all, the nomogram we constructed demonstrated superior discriminative ability and accuracy when compared to the 8th AJCC TNM staging system.

### Risk stratification for survival analysis

**Figure 6:**
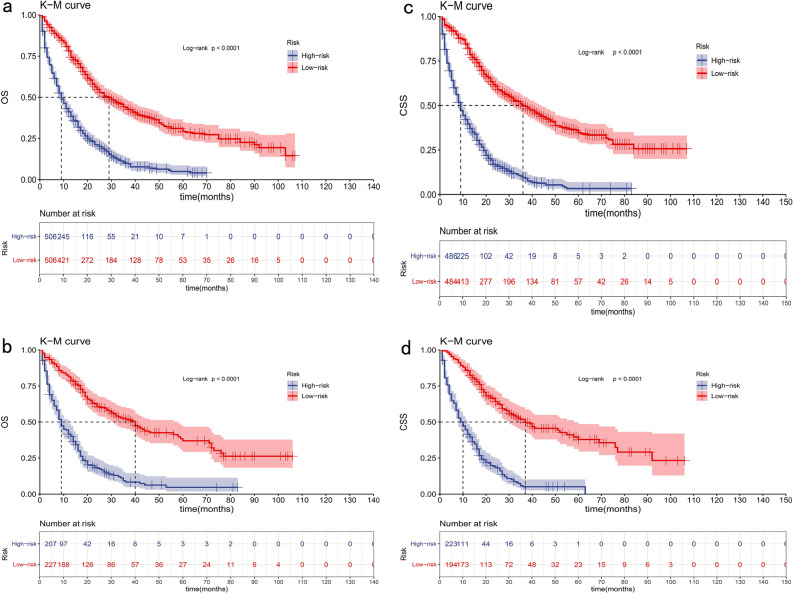
Kaplan–Meier curves for ICCA patients in the low- and high-risk groups in the training cohort (**a**) and validation cohort (**b**) for the prediction of OS. Kaplan–Meier curves for patients in the low- and high-risk groups in the training cohort (**c**) and validation cohort (**d**) for the prediction of CSS.

In the OS group, according to the total points derived from the nomogram in the training cohort, we divided patients into high-risk and low-risk groups based on the average score (total points over 187 or under 187) and conducted a survival analysis (Fig. 6a,b). In the training cohort, 506 patients were classified as the low-risk group, with a median OS of 29 (95% CI 24.36–33.65) months. On the other hand, 506 patients were assigned to the high-risk group, with a median OS of 9 (95% CI 7.73–10.27) months. Similarly, the validation cohort was categorized into high-risk and low-risk groups based on the score threshold determined by the training cohort, with the median OS as 9 (95% CI 7.45–10.55) months and 40 (95% CI 31.65–48.35) months. The differences in KM curves between high-risk and low-risk groups are all statistically significant in both cohorts (P < 0.0001).

In the CSS group, high-risk and low-risk groups were separated by the score threshold of 162, and survival analysis was similarly performed (Fig. 6c,d). In the training cohort, the low-risk group has a median CSS of 36 months (95% CI 29.80–42.20), while the high-risk group has a median CSS of 9 months (95% CI 7.86–10.14). In the validation cohort, the high-risk group has a median CSS of 10 months (95% CI 8.06–11.94) and the low-risk group has a median CSS of 37 months (95% CI 22.37–51.63). The differences between high-risk and low-risk groups are statistically significant (P < 0.0001).

### Subgroup analysis for ICCA patients without surgery

**Figure 7:**
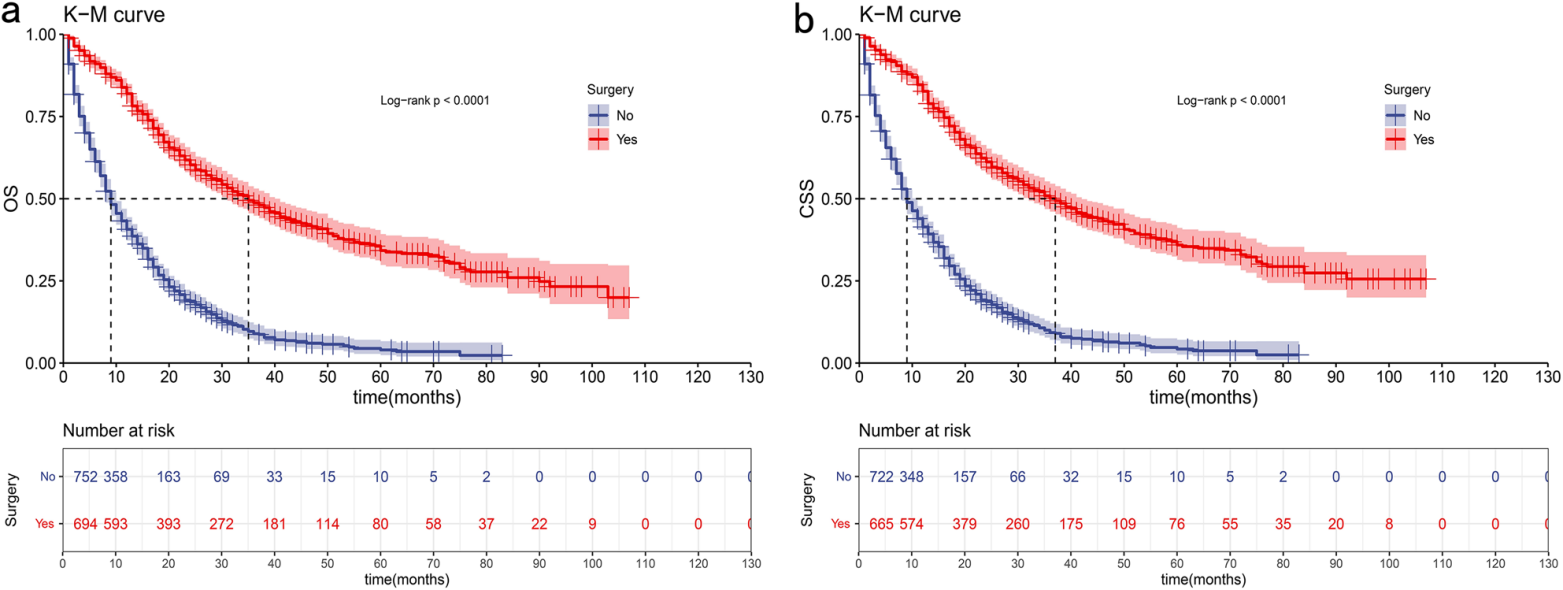
Kaplan–Meier curves for ICCA patients with or without surgery for the prediction of OS (**a**) and CSS (**b**).

In both the OS and CSS group, more than half of patients could not undergo surgery for primary treatment (52.0% and 52.1% in the OS group and CSS group separately). While surgical status constitutes a significant proportion of our nomogram model. By conducting a survival analysis of all eligible patients in the OS and CSS group, stratified by surgical status, we identified a significant difference in the prognosis between patients who underwent surgery and those who did not (P < 0.0001) (Fig. 7a,b). Therefore, we conducted a subgroup analysis for the patients without surgery and developed another two nomograms to predict the OS and CSS of these patients.

In the OS analysis group, there were 752 eligible patients. The training (n = 526) and validation (n = 226) cohort was randomly generated from these patients with a proportion of 7:3. In the CSS analysis group, excluding 30 patients who died due to other causes of death, a total of 722 patients were included, including 505 patients from the training cohort and 217 patients from the validation cohort. The demographic and clinical characteristics of the OS group and CSS group were presented in Tables 6 and 7 separately. It is noteworthy that the median survival time of patients in the non-surgical group significantly shortened (P < 0.0001).

**Table 6:** Demographic and clinical characteristics of patients with intrahepatic cholangiocarcinoma without surgery in the OS group. *IQR* interquartile range.

Characteristic	Overall	Training cohort	Validation cohort	P value
Total patients, n	752	526	226	
Age, n (%)				0.277
< 50	76 (10.1%)	59 (11.2%)	17 (7.5%)	
50–64	278 (37.0%)	185 (35.2%)	93 (41.2%)	
65–79	325 (43.2%)	231 (43.9%)	94 (41.6%)	
≥ 80	73 (9.7%)	51 (9.7%)	22 (9.7%)	
Sex, n (%)				0.713
Male	397 (52.8%)	280 (53.2%)	117 (51.8%)	
Female	355 (47.2%)	246 (46.8%)	109 (48.2%)	
Race, n (%)				0.284
White	596 (79.3%)	409 (77.8%)	187 (82.7%)	
Black	64 (8.5%)	47 (8.9%)	17 (7.5%)	
Other	92 (12.2%)	70 (13.3%)	22 (9.7%)	
AJCC T stage, n (%)				0.484
T1	199 (26.5%)	144 (27.4%)	55 (24.3%)	
T2	388 (51.6%)	264 (50.2%)	124 (54.9%)	
T3	115 (15.3%)	85 (16.2%)	30 (13.3%)	
T4	50 (6.6%)	33 (6.3%)	17 (7.5%)	
AJCC N stage, n (%)				0.980
N0	462 (61.4%)	323 (61.4%)	139 (61.5%)	
N1	290 (38.6%)	203 (38.6%)	87 (38.5%)	
AJCC M stage, n (%)				0.288
M0	444 (59.0%)	304 (57.8%)	140 (61.9%)	
M1	308 (41.0%)	222 (42.2%)	86 (38.1%)	
Grade, n (%)				0.568
I	72 (9.6%)	54 (10.3%)	18 (8.0%)	
II	306 (40.7%)	210 (39.9%)	96 (42.5%)	
III + IV	374 (49.7%)	262 (49.8%)	112 (49.5%)	
Tumor size, n (%)				0.009
≤ 5 cm	183 (24.3%)	142 (27.0%)	41 (18.1%)	
> 5 cm	569 (75.7%)	384 (73.0%)	185 (81.9%)	
Chemotherapy, n (%)				0.298
No	236 (31.4%)	159 (30.2%)	77 (34.1%)	
Yes	516 (68.6%)	367 (69.8%)	149 (65.9%)	
Radiotherapy, n (%)				0.845
No	622 (82.7%)	436 (82.9%)	186 (82.3%)	
Yes	130 (17.3%)	90 (17.1%)	40 (17.7%)	
Survival, median (IQR)	9 (3.0, 18.00)	9 (4.0, 17.00)	8 (3.0, 18.00)	0.758

**Table 7:** Demographic and clinical characteristics of patients with Intrahepatic cholangiocarcinoma without surgery in the CSS group. *IQR* interquartile range.

Characteristic	Overall	Training cohort	Validation cohort	P value
Total patients, n	722	505	217	
Age, n (%)				0.332
< 50	76 (10.5%)	59 (11.1%)	17 (7.8%)	
50–64	272 (37.7%)	182 (37.0%)	90 (41.5%)	
65–79	308 (42.7%)	217 (42.6%)	91 (41.9%)	
≥ 80	66 (9.1%)	47 (9.3%)	19 (8.8%)	
Sex, n (%)				0.695
Male	384 (53.2%)	271 (53.7%)	113 (52.1%)	
Female	338 (46.8%)	234 (46.3%)	104 (47.9%)	
Race, n (%)				0.278
White	574 (79.5%)	394 (78.0%)	180 (82.9%)	
Black	62 (8.6%)	45 (8.9%)	17 (7.8%)	
Other	86 (11.9%)	66 (13.1%)	20 (9.2%)	
AJCC T stage, n (%)				0.524
T1	183 (25.3%)	132 (26.1%)	51 (23.5%)	
T2	379 (52.5%)	260 (51.5%)	119 (54.8%)	
T3	113 (15.7%)	83 (16.4%)	30 (13.8%)	
T4	47 (6.5%)	30 (5.9%)	17 (7.8%)	
AJCC N stage, n (%)				0.953
N0	438 (60.7%)	306 (60.6%)	132 (60.8%)	
N1	284 (39.3%)	199 (39.4%)	85 (39.2%)	
AJCC M stage, n (%)				
M0	422 (58.4%)	290 (57.4%)	132 (60.8%)	0.395
M1	300 (41.6%)	215 (42.6%)	85 (39.2%)	
Grade, n (%)				0.421
I	69 (9.6%)	53 (10.5%)	16 (7.4%)	
II	293 (40.6%)	202 (40.0%)	91 (41.9%)	
III + IV	360 (49.9%)	250 (49.5%)	110 (50.7%)	
Tumor size, n (%)				0.012
≤ 5 cm	170 (23.5%)	132 (26.1%)	38 (17.5%)	
> 5 cm	552 (76.5%)	373 (73.9%)	179 (82.5%)	
Chemotherapy, n (%)				0.270
No	222 (30.7%)	149 (29.5%)	73 (33.6%)	
Yes	500 (69.3%)	356 (70.5%)	144 (66.4%)	
Radiotherapy, n (%)				0.661
No	599 (83.0%)	421 (83.4%)	178 (82.0%)	
Yes	123 (17.0%)	84 (16.6%)	39 (18.0%)	
Survival, median (IQR)	9 (3.0, 18.00)	9 (4.0, 17.50)	9 (3.0, 18.00)	0.687

In both the OS and CSS groups, by performing univariable and multivariable Cox proportional hazards regression analyses, we screened out the same five independent prognostic factors for the nomogram construction: sex, AJCC T stage, AJCC M stage, tumor grade, and chemotherapy status. The detailed information on univariable and multivariable Cox regression analyses in the OS and CSS group is shown in Supplementary Table 1 and Supplementary Table 2 accordingly.

**Figure 8:**
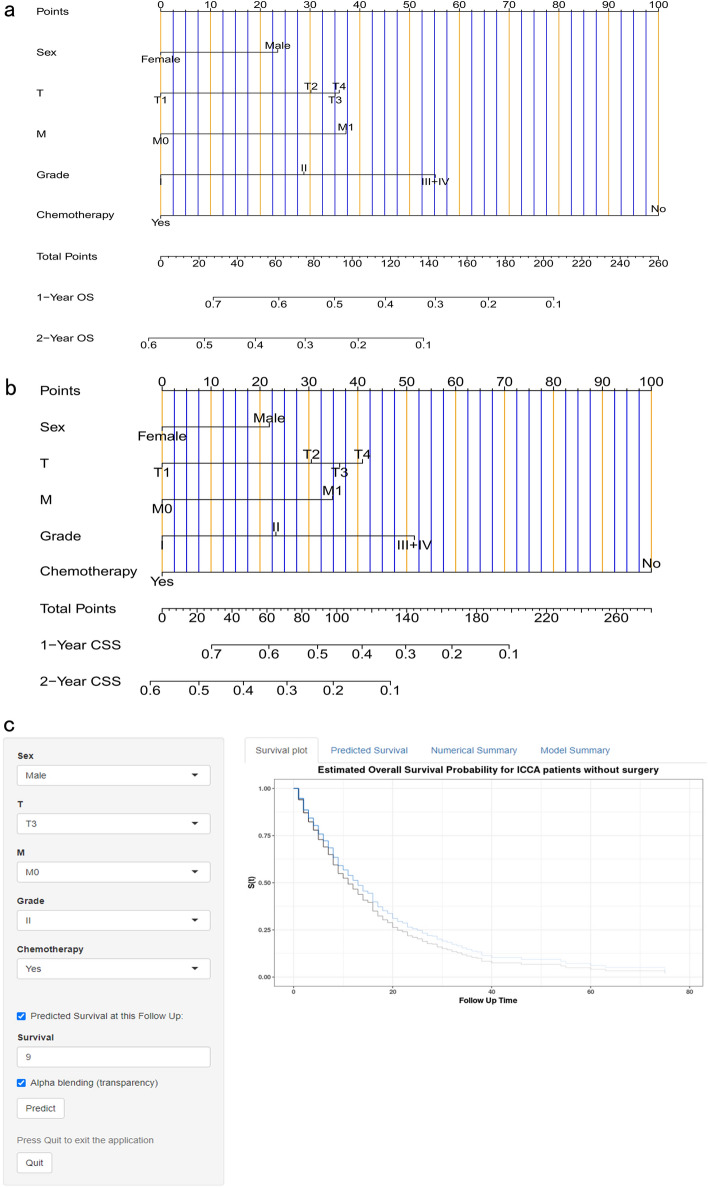
(**a**) Nomogram predicting 1-, and 2-year OS for ICCA patients without surgery. (**b**) Nomogram predicting 1-, and 2-year CSS for ICCA patients without surgery. (**c**) A web-based dynamic nomogram predicting OS probabilities for ICCA patients without surgery (available at: https://tumornomogram.shinyapps.io/Intrahepatic_cholangiocarcinoma_OS_a/).

Differing from the overall population, patients in the non-surgical group experienced a significantly shortened survival time (P < 0.0001). In the training cohort of the OS group, only 1.3% of the patients reached the OS of 5 years and 5.5% reached the OS of 3 years. In the training cohort of the CSS group, the corresponding proportions were 1.4% and 5.7% respectively. Based on patient survival status and five prognostic factors, we developed two nomograms using the multivariate Cox prognostic model for ICCA patients without surgery which enabled the prediction of 1-, and 2-year OS and CSS rates (Fig. 8a,b). To validate the nomogram, we calculated the C-index, AUC of ROC curves, drew calibration curves, and performed the DCA analysis in each cohort.

In the OS group, The C-index of the nomogram in the training and validation cohorts were 0.676 (0.644–0.709) and 0.700 (0.650–0.750) respectively. As a comparison, the C-index of the 8th AJCC staging was 0.544 (0.514–0.575) and 0.597 (0.549–0.650) in the training and validation cohorts. In the CSS group, The C-index of the nomogram in the training and validation cohorts were 0.680 (0.647–0.713) and 0.694 (0.642–0.745). In comparison, the C-index of the 8th AJCC staging was 0.547 (0.519–0.578) and 0.600 (0.551–0.649) in the training and validation cohorts. Calibration curves of the nomogram for predicting the 1-, and 2-year OS and CSS rates in both cohorts showed good agreement with survival status (Supplementary Fig. 2a–h).

**Figure 9:**
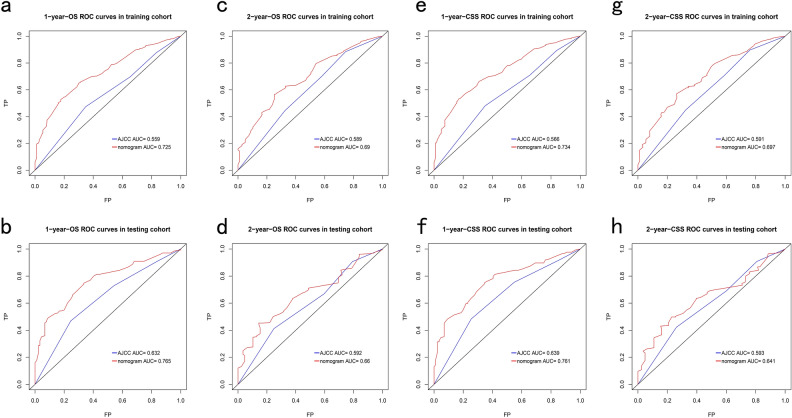
ROC curves of both the nomogram and the 8th AJCC TNM staging system in the training cohort (**a**,**b**) and the validation cohort (**c**,**d**) for 1-, and 2-year OS prediction of ICCA patients without surgery. ROC curves of both the nomogram and the 8th AJCC TNM staging system in the training cohort (**e**,**f**) and the validation cohort (**g**,**h**) for 1-, and 2-year CSS prediction of ICCA patients without surgery.

Time-dependent ROC curves and DCA curves were plotted to compare the performance of the nomogram with the 8th AJCC staging system, and all of the AUC values surpassed the corresponding AUC values of the AJCC stage in both the OS and CSS group. Figure 9a–h exhibited the AUC of the nomogram and the AJCC stage in each ROC curve. More detailed parameters of the nomogram are listed in Table 8. The AUC for the prediction of 1-year survival in both the OS and CSS groups was higher than the prediction of 2-year survival.

**Table 8:** Parameters of the ROC curves for the prediction of OS and CSS of intrahepatic cholangiocarcinoma patients without surgery. *AUC* areas under curve, *Se* sensitivity, *Sp* specificity, *PPV* positive predictive value.

Training cohort	AUC	Se/Sp	PPV (%)	Validation cohort	AUC	Se/Sp	PPV (%)
For 1-year OS	0.725	53.31%/83.17%	81.28	For 1-year OS	0.765	73.80%/66.67%	76.31
For 2-year OS	0.690	55.42%/76.62%	91.25	For 2-year OS	0.660	45.12%/87.10%	93.62
For 1-year CSS	0.734	53.86%/83.82%	81.46	For 1-year CSS	0.761	74.15%/65.48%	75.51
For 2-year CSS	0.697	55.88%/75.68%	90.91	For 2-year CSS	0.641	42.84%/86.21%	92.95

DCA analyses in both cohorts were performed to exhibit the net benefit of the nomogram compared to the AJCC stage, and the nomogram exhibited a greater net benefit when predicting the 1-, and 2-year OS and CSS rates compared to the 8th AJCC TNM staging system (Supplementary Fig. 3a–h).

**Figure 10:**
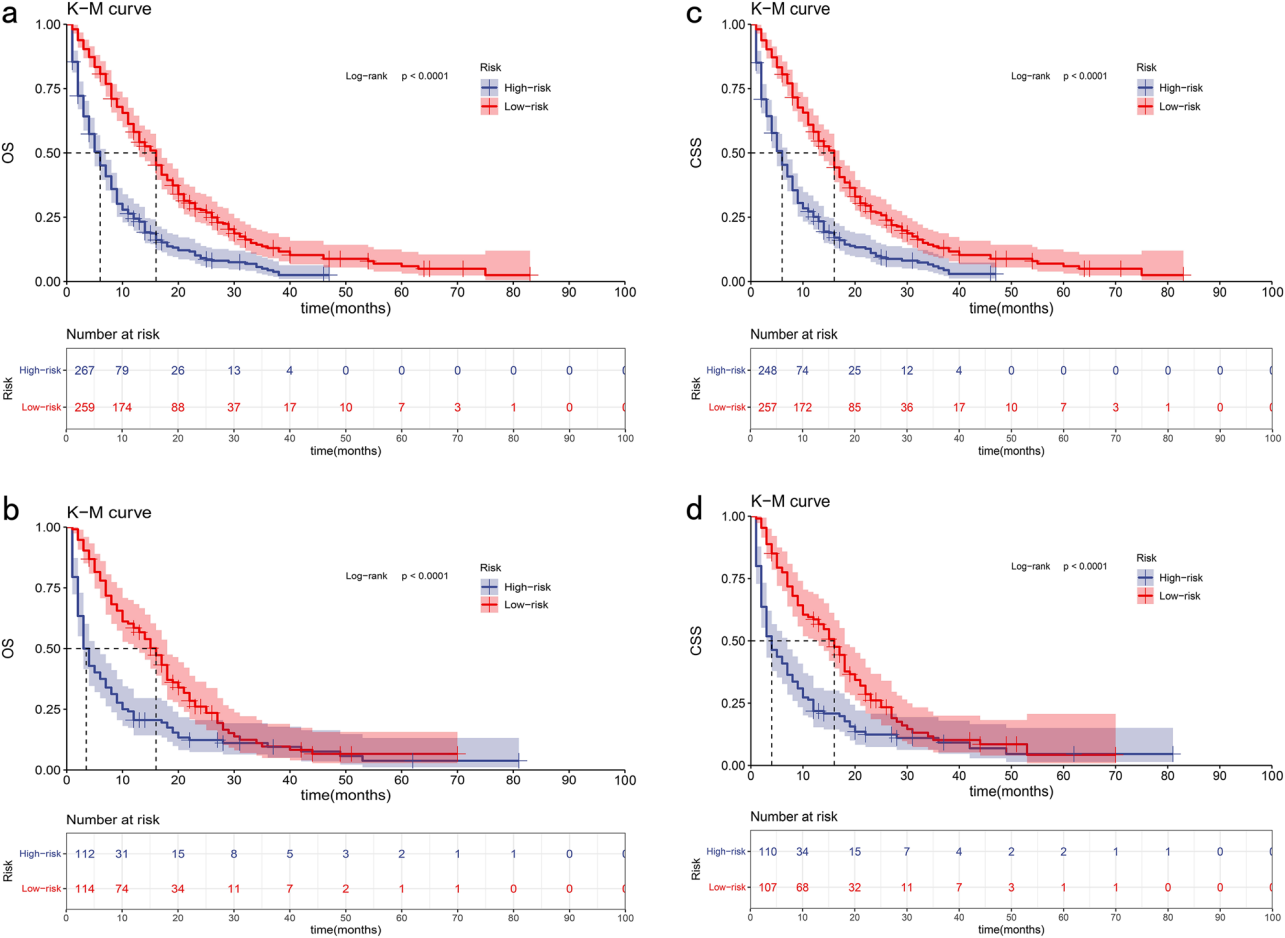
Kaplan–Meier curves for ICCA patients without surgery in the low- and high-risk groups in the training cohort (**a**) and validation cohort (**b**) for the prediction of OS. Kaplan–Meier curves for patients in the low- and high-risk groups in the training cohort (**c**) and validation cohort (**d**) for the prediction of CSS.

Survival analyses were performed according to the risk score. In the training cohort of the OS group, among the 259 patients categorized as the low-risk group with a score below 116, the median survival was 16 months (95% CI 14.16–17.84). The rest 267 patients were assigned to the high-risk group, with a median survival of 6 (95% CI 4.90–7.10) months. In the validation cohort of the OS group, the median survival time was 16 (95% CI 13.23–18.77) months and 3 (95% CI 2.10–3.90) months accordingly. In the training cohort of the CSS group, of the 257 patients with a score below 109 identified in the low-risk group, the median survival was 16 (95% CI 14.22–17.78) months. Conversely, the high-risk group comprised 248 patients, with a median survival of 6 (95% CI 4.89–7.11) months. In the validation cohort of the CSS group, the median survival time was 16 (95% CI 12.88–19.12) months and 4 (95% CI 2.60–5.40) months accordingly. The differences in KM curves between high-risk and low-risk groups were all statistically significant in both cohorts (P < 0.0001) (Fig. 10a–d).

### Nomogram publication

After performing the validation and comparative evaluation analysis aforementioned, our nomograms have demonstrated reliability. To further assist researchers in conveniently predicting ICCA patient prognosis, we published four web-based nomograms (https://tumornomogram.shinyapps.io/Intrahepatic_cholangiocarcinoma_OS/, https://tumornomogram.shinyapps.io/Intrahepatic_cholangiocarcinoma_OS_a/, https://tumornomogram.shinyapps.io/Intrahepatic_cholangiocarcinoma_CSS/, and https://tumornomogram.shinyapps.io/Intrahepatic_cholangiocarcinoma_CSS_a/) which could allow for the dynamic visualization of one individual patient’s prognostic outcome. Figure 8c provides an overview of one web-based nomogram for the prediction of OS probabilities for ICCA patients without surgery.

## Discussion

ICCA is one of the most common malignant tumors in elderly patients, with an average age of diagnosis around 70 years^22^. Both the incidence and mortality of patients with ICCA have been increasing steadily over the past 2 decades^4^. The challenges in early diagnosis of ICCA and the limited availability of radical surgical options further impact the prognosis of patients with ICCA. Therefore, one specifically developed prognostic prediction model for ICCA patients was required to assist researchers in accurately assessing prognosis and facilitating informed treatment decisions. In our study, we constructed four nomograms to predict the OS and CSS of ICCA patients depending on the surgical status of patients, and after validation, the nomograms demonstrated robust discriminative performance and calibration. Moreover, the risk stratification exhibited a commendable capability to distinguish ICCA patients into high- and low-risk groups.

Currently, the most widely used 8th edition of the AJCC staging system was imprecise in prognosis evaluation^23^. In comparison to the AJCC staging system, the development of a nomogram allows for the inclusion of additional risk factors including age, sex, and treatment status, enabling more precise prognoses. Several researchers have utilized nomograms solely for the prediction of OS in patients with ICCA^9–11^, without considering non-cancer-specific mortality. This oversight is particularly significant when considering the substantial population of elderly patients. Some previously published nomograms predicting CSS in ICCA patients concentrated more on the survival status after surgery^12,13^, which also excluded a significant number of patients who were not able to undergo surgical treatment and restricted the scope of their applicability. Zhao et al. previously conducted a population‑based analysis to build a prognostic model for CSS prediction in ICCA patients^24^. In contrast to our study, their investigation encompassed a broader spectrum of morphological categories, encompassing spindle cell carcinoma, squamous cell carcinoma, adenocarcinoma, scirrhous adenocarcinoma, and others, rather than being limited to cholangiocarcinoma as typically observed in most relevant research studies, which may result in a reduction in the accuracy of the model. Above all, in the current landscape of prognostic research on ICCA, there is a lack of comprehensive studies on both the OS and CSS analysis. Our study addressed this gap in the field. Additionally, we have embarked on pioneering exploration for the subset of patients who could not undergo surgery.

In the univariable regression analysis of both the OS and CSS group, age, sex, AJCC T stage, AJCC N stage, AJCC M stage, surgery, tumor grade, and tumor size were potential prognostic factors for ICCA patients. After conducting subsequent multivariate regression analysis, it was revealed that the rest seven factors, excluding tumor size, were identified as independent prognostic factors for the development of the nomogram. In both groups, the predictive capability of the nomogram surpassed that of AJCC staging. In addition, the C-index levels between the training set and validation set remained consistent, reflecting the reliability of the nomograms.

Similar findings were observed in the ROC analysis that the nomogram demonstrated superior performance compared to the AJCC staging system (Fig. 4a–l). A widely accepted criterion for determining the discriminative ability of a model is to consider it relatively good if its C-index and AUC surpass the threshold of 0.7^25^. Therefore, our nomogram exhibits a robust discriminatory capacity. In addition, the calibration plots aligned closely with the diagonal slash, indicating excellent calibration of our nomograms^26^. In the DCA analysis to evaluate the net benefits of different risk thresholds, our nomogram exhibited higher clinical benefits than the AJCC stage in predicting the OS and CSS of patients with ICCA.

Notably, our research found that surgical status had the most significant weight in both nomograms. For ICCA patients, surgery remained the mainstream of the treatment which may prolong a median disease‐free survival up to 34 months^27^. The primary reasons for unresectable patients include multiple tumors, nodal or peritoneal metastases, and advanced hepatic disease^5^. For patients with unresectable and metastatic ICCA, chemotherapy with a combination of gemcitabine and cisplatin was recommended as the primary treatment with a median OS of 11.7 months^28^. One recent study has shown promising results for utilizing the nanoparticle albumin-bound-paclitaxel plus gemcitabine-cisplatin regimen in converting patients who are not eligible for surgery into viable candidates for surgical intervention^29^. Another recent study has confirmed that when chemotherapy is combined with toripalimab and lenvatinib, it exhibited potential for conversion therapy^30^. Therefore, although chemotherapy was not one of the independent predictors in the OS and CSS group, we should take note of the contributions of chemotherapy-based treatment in conversion therapy.

Considering that more than half of the patients could not receive surgery as primary treatment in our whole cohort, and coupled with the substantial weight attributed to the surgical status in our results, we performed a subgroup analysis for patients without surgery to explore factors influencing the prognosis of these patients. Constrained by the unfavorable prognosis of patients unable to undergo surgery, our predicted survival time was limited to 1 year and 2 years. We performed two nomograms for the prediction of the OS and CSS accordingly, and both the nomograms contained the same five independent predictive factors: sex, AJCC T stage, AJCC M stage, tumor grade, and chemotherapy. Chemotherapy had the highest contribution to both nomograms. This aligned with the current treatment recommendations for advanced-stage ICCA, advocating for an active systemic therapy centered around chemotherapy. Although the predictive capabilities of both nomograms surpassed the AJCC staging system, they exhibited weaker performance in predicting 2-year OS and CSS compared to their 1-year predictions. The AUC of the nomogram for predicting 2-year OS and CSS in the training cohort was 0.690 and 0.697 respectively, not surpassing 0.7. We attributed this to the limited sample size of patients achieving a 2-year prognosis. However, our nomogram still demonstrated high accuracy in predicting 1-year survival.

In our study, we incorporated various factors that influenced the OS and CSS of ICCA patients from both demographic and treatment perspectives. While confirming the prognostic value of AJCC staging in patients with ICCA, we have also affirmed the benefits of surgical treatment and chemotherapy for patient prognosis. Therefore, we recommended that ICCA patients should be further divided into high-risk and low-risk groups according to the nomogram risk score to better differentiate the distinct prognoses between the two groups of patients. In our survival analysis, there were significant differences in the median survival time between high-risk and low-risk groups. Therefore, early identification of patients' prognostic status holds crucial guidance for implementing more personalized treatment strategies. For example, if one patient could not receive surgery and was categorized into the high-risk group, based on the currently published clinical survival time and prognosis benefit outcomes, selecting proper clinical trials based on genetic testing results may lead to more effective treatment outcomes. Furthermore, we developed user-friendly web-based predictive tools aimed at facilitating physicians in conducting patient prognosis assessments and implementing personalized treatment plans more conveniently.

Undeniably, this study still has shortcomings. Firstly, this is a retrospective study which may contain selection bias due to the lack of clinical data. Secondly, data extracted from the SEER database lacked information on immunotherapy and targeted therapy, which could effectively improve patient survival. Thirdly, the SEER database only included patients from the United States where the Asian population represented only a small fraction. Whether the result could be utilized in populations of different races, especially in Southeast Asian countries where the incidence of ICCA was much higher than HCC still needs further research^4^. Finally, in the nomograms, the weight of surgery and chemotherapy are the highest, but radiation is not included in the nomogram. Although nomogram could apply to predict the prognosis, we should not overlook the positive impact of other treatment modalities, including radiotherapy, on patient prognosis. The recommendation of treatment modality should be seriously treated.

## Conclusion

In summary, the nomograms which included age, sex, AJCC T, N, M stage, surgery status, and tumor grade as independent prognostic factors for predicting 1-, 3-, and 5-year OS and CSS rates in ICCA patients performed better than the traditional AJCC staging system. For patients who could not receive surgery as primary treatment, the nomograms which included sex, AJCC T, M stage, tumor grade, and chemotherapy as independent prognostic factors for predicting 1-, and 2-year OS and CSS rates also performed better than the AJCC staging system. Notably, surgical status contributed most to the OS and CSS nomograms, and for patients without surgery, chemotherapy contributed most to the OS and CSS prediction. These novel nomograms could enable more accurate prognostic prediction and help clinicians provide better-personalized healthcare.

### Supplementary Information

Supplementary Information.

## Data Availability

The original contributions presented in the study are included in the article. Further inquiries can be directed to the corresponding author. Publicly available datasets of this study could be acquired from: https://seer.cancer.gov/.
