# Supplementary material for: Development and validation of web-based nomograms for predicting survival status in patients with intrahepatic cholangiocarcinoma depending on the surgical status: a SEER database analysis
Source: Sci Rep. 2024 Jan 18;14:1568. doi: 10.1038/s41598-024-52025-3 (PMC10796320; doi:10.1038/s41598-024-52025-3)
Supplement: Supplementary file 1 — Supplementary Information. [file 41598_2024_52025_MOESM1_ESM.docx]

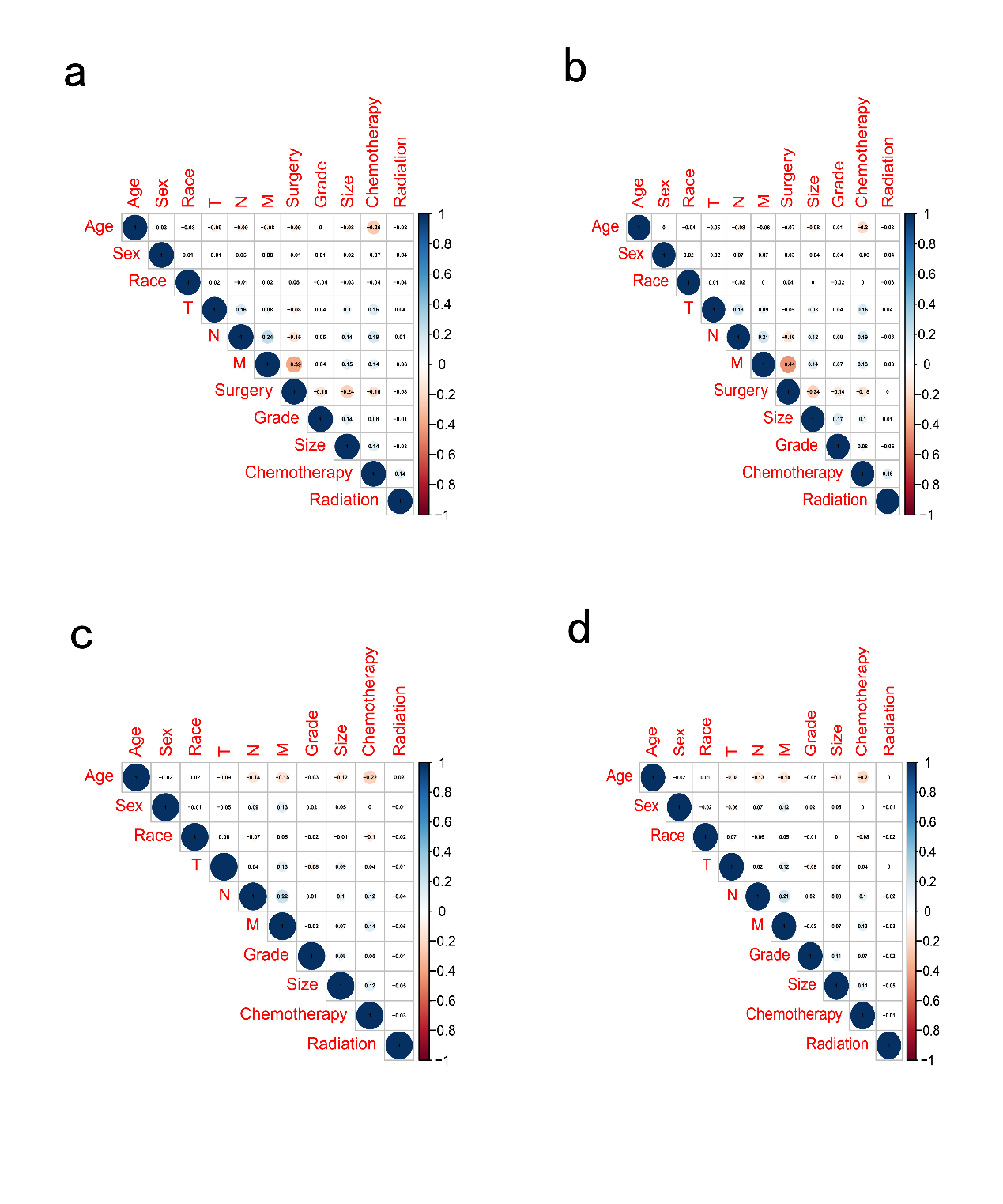


**Supplementary Figure 1. The results of correlation analysis between all included variables. (a) The OS group. (b) The CSS group. (c) The non-surgery group for OS analysis. (d) The non-surgery group for CSS analysis.**

**
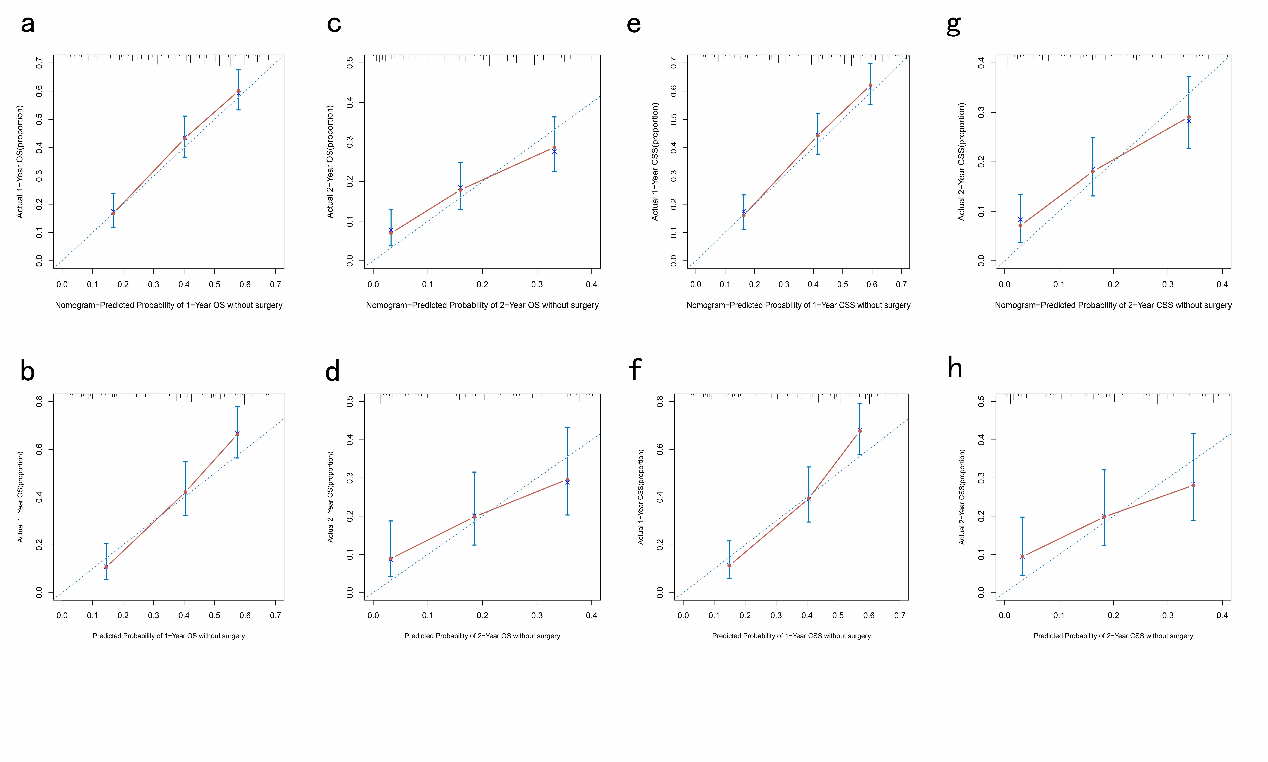
**

**Supplementary Figure 2. Calibration curves of the nomogram for the 1-, and 2-year OS prediction of patients with ICCA without surgery in the** **training cohort (a, c) and the validation cohort (b, d). Calibration curves of the nomogram for the 1-, and 2- CSS prediction of patients with ICCA without surgery in the** **training cohort (e, g) and the validation cohort (f, h).**

**
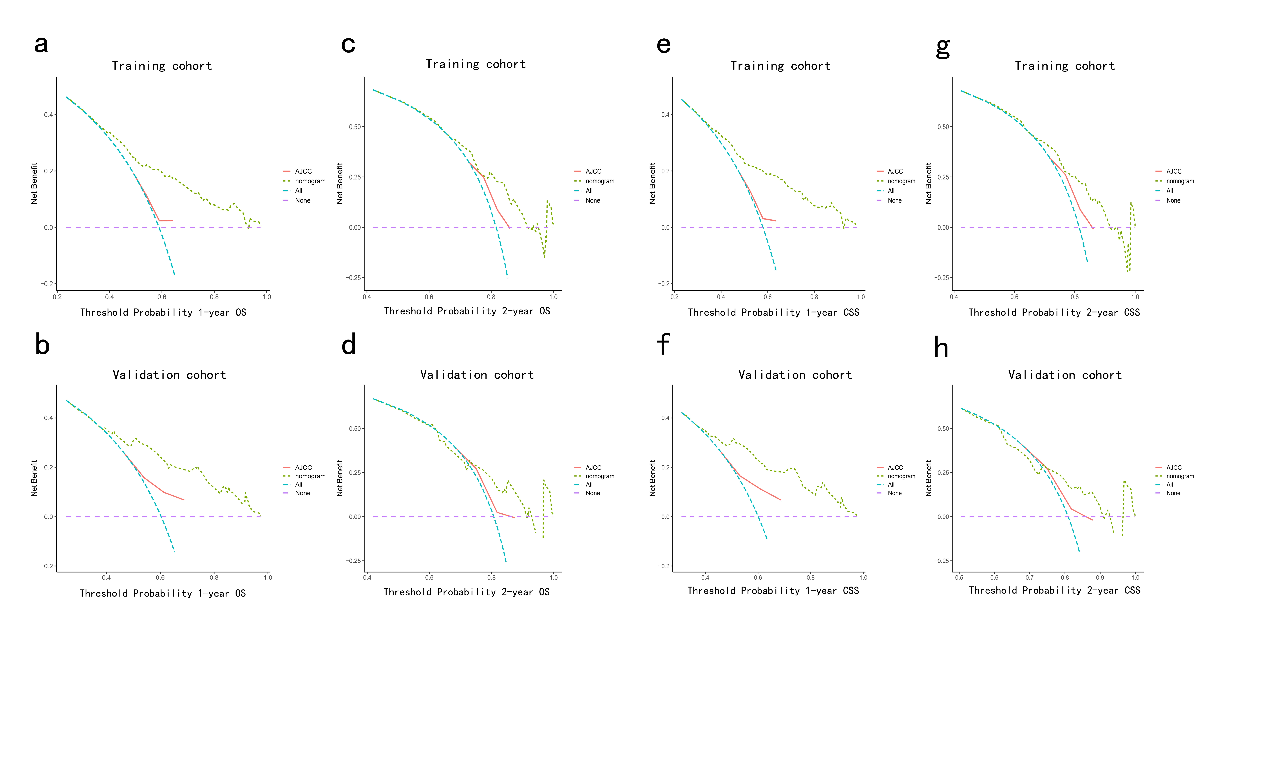
**

**Supplementary Figure 3. Decision curves of both the nomogram and the 8th AJCC TNM staging system in the training cohort (a, c) and the validation cohort (b, d) for 1-, and 2-year OS prediction of ICCA patients without surgery. Decision curves of both the nomogram and the 8th AJCC TNM staging system in the training cohort (e, g) and the validation cohort (f, h) for 1-, and 2-year CSS prediction of ICCA patients without surgery.**

| **Variables** | **Univariable Cox analysis** | | **Multivariable Cox analysis** | |
| --- | --- | --- | --- | --- |
|  | **HR (95% CI)** | **P value** | **HR (95% CI)** | **P value** |
| **Age** | | | | |
| **<50** | Reference | | Reference | |
| **50-64** | 1.275(0.927-1.752) | 0.135 | 1.290(0.937-1.776) | 0.119 |
| **65-79** | 1.319(0.967-1.798) | 0.080 | 1.333(0.974-1.826) | 0.073 |
| **≥80** | 1.621(1.087-2.418) | 0.018* | 1.318(0.855-2.032) | 0.211 |
| **Sex** | | | | |
| **Female** | Reference | | Reference | |
| **Male** | 1.249(1.039-1.501) | 0.018* | 1.252(1.038-1.511) | 0.019* |
| **Race** | | | | |
| **White** | Reference | |  | |
| **Black** | 1.187(0.858-1.641) | 0.301 |  |  |
| **Other** | 1.146(0.876-1.498) | 0.319 |  |  |
| **AJCC T stage** | | | | |
| **T1** | Reference | | Reference | |
| **T2** | 1.255(1.007-1.563) | 0.043* | 1.380(1.095-1.740) | 0.006** |
| **T3** | 1.328(0.993-1.776) | 0.055 | 1.462(1.079-1.981) | 0.014* |
| **T4** | 1.182(0.787-1.776) | 0.420 | 1.402(0.925-2.127) | 0.112 |
| **AJCC N stage** | | | | |
| **N0** | Reference | |  | |
| **N1** | 1.183(0.981-1.426) | 0.078 |  |  |
| **AJCC M stage** | | | | |
| **M0** | Reference | | Reference | |
| **M1** | 1.263(1.051-1.519) | 0.013* | 1.461(1.198-1.783) | <0.001*** |
| **Grade** | | | | |
| **I** | Reference | | Reference | |
| **II** | 1.204(0.863-1.682) | 0.275 | 1.355(0.964-1.905) | 0.08 |
| **III+IV** | 1.479(1.068-2.049) | 0.019* | 1.763(1.265-2.457) | <0.001*** |
| **Tumor size** | | | | |
| **≤5cm** | Reference | |  | |
| **>5cm** | 0.984(0.801-1.209) | 0.878 |  |  |
| **Chemotherapy** | | | | |
| **No** | Reference | | Reference | |
| **Yes** | 0.458 (0.375-0.559) | <0.001*** | 0.375(0.300-0.468) | <0.001*** |
| **Radiotherapy** | | | | |
| **No** | Reference | |  | |
| **Yes** | 0.819(0.641-1.047) | 0.111 |  |  |

**Supplementary Table 1. Univariable and multivariable Cox analyses on variables for the prediction of OS of intrahepatic cholangiocarcinoma patients without surgery. *HR* hazard ratio, *CI* confidence interval, *p<0.05, **p<0.01, ***p<0.001.**

| **Variables** | **Univariable Cox analysis** | | **Multivariable Cox analysis** | |
| --- | --- | --- | --- | --- |
|  | **HR (95% CI)** | **P value** | **HR (95% CI)** | **P value** |
| **Age** | | | | |
| **<50** | Reference | | Reference | |
| **50-64** | 1.276(0.928-1.756) | 0.134 | 1.285(0.933-1.771) | 0.125 |
| **65-79** | 1.271(0.931-1.737) | 0.132 | 1.295(0.944-1.776) | 0.109 |
| **≥80** | 1.732 (1.150-2.609) | 0.009** | 1.437(0.926-2.232) | 0.106 |
| **Sex** | | | | |
| **Female** | Reference | | Reference | |
| **Male** | 1.250(1.035-1.509) | 0.021* | 1.243(1.026-1.505) | 0.026* |
| **Race** | | | | |
| **White** | Reference | |  | |
| **Black** | 1.226(0.880-1.710) | 0.229 |  |  |
| **Other** | 1.144(0.867-1.508) | 0.342 |  |  |
| **AJCC T stage** | | | | |
| **T1** | Reference | | Reference | |
| **T2** | 1.286(1.023-1.615) | 0.031* | 1.418(1.115-1.802) | 0.004** |
| **T3** | 1.353(1.003-1.824) | 0.048* | 1.519(1.110-2.079) | 0.009** |
| **T4** | 1.174(0.764-1.805) | 0.463 | 1.520(0.979-2.361) | 0.062 |
| **AJCC N stage** | | | | |
| **N0** | Reference | |  | |
| **N1** | 1.192(0.985-1.443) | 0.071 |  |  |
| **AJCC M stage** | | | | |
| **M0** | Reference | | Reference | |
| **M1** | 1.270(1.052-1.534) | 0.013* | 1.454(1.188-1.780) | <0.001*** |
| **Grade** | | | | |
| **I** | Reference | | Reference | |
| **II** | 1.165(0.830-1.634) | 0.377 | 1.311(0.929-1.851) | 0.123 |
| **III+IV** | 1.438(1.034-2.001) | 0.031* | 1.761(1.257-2.467) | <0.001*** |
| **Tumor size** | | | | |
| **≤5cm** | Reference | |  | |
| **>5cm** | 1.012(0.818-1.252) | 0.911 |  |  |
| **Chemotherapy** | | | | |
| **No** | Reference | | Reference | |
| **Yes** | 0.435(0.353-0.535) | <0.001*** | 0.363(0.289-0.457) | <0.001*** |
| **Radiotherapy** | | | | |
| **No** | Reference | |  | |
| **Yes** | 0.805(0.624-1.039) | 0.095 |  |  |

**Supplementary Table 2. Univariable and multivariable Cox analyses on variables for the prediction of CSS of intrahepatic cholangiocarcinoma patients without surgery. *HR* hazard ratio, *CI* confidence interval, *p<0.05, **p<0.01, ***p<0.001.**
